# *Harryplax severus*, a new genus and species of an unusual coral rubble-inhabiting crab from Guam (Crustacea, Brachyura, Christmaplacidae)

**DOI:** 10.3897/zookeys.647.11455

**Published:** 2017-01-23

**Authors:** Jose C. E. Mendoza, Peter K. L. Ng

**Affiliations:** 1 Lee Kong Chian Natural History Museum, Faculty of Science, National University of Singapore, 2 Conservatory Drive, 117377 Singapore

**Keywords:** Pacific, Mariana Islands, taxonomy, Decapoda, Pseudozioidea, coral reef, coelobite, cryptofauna

## Abstract

*Harryplax severus*, a new genus and species of coral rubble-dwelling pseudozioid crab is described from the island of Guam in the western Pacific Ocean. The unusual morphological features of its carapace, thoracic sternum, eyes, antennules, pereopods and gonopods place it in the family Christmaplacidae Naruse & Ng, 2014. A suite of characters on the cephalothorax, pleon and appendages distinguishes *Harryplax severus* **gen. & sp. n.** from the previously sole representative of the family, *Christmaplax mirabilis* Naruse & Ng, 2014, described from Christmas Island in the eastern Indian Ocean. This represents the first record of Christmaplacidae in the Pacific Ocean. With the discovery of a second genus, a revised diagnosis for Christmaplacidae is provided.

## Introduction

Naruse and Ng (2014) described a new genus and new species of pseudozioid crab, *Christmaplax mirabilis*, from an anchialine cave in Christmas Island, an Australian territory in the eastern Indian Ocean. They argued that there were sufficient unique suprageneric characters to distinguish this species from the remainder of the Pseudozioidea, necessitating the establishment of a new family, Christmaplacidae. In addition to the establishment of this family and the clarification of familial morphological characters within Pseudozioidea, Naruse and Ng (2014) also transferred *Flindersoplax* Davie, 1989, previously in Pseudoziidae sensu Ng et al. (2008) to the Planopilumnidae Ng, 2010, on the basis of the characters of the male anterior thoracic sternum and pleon and the G1.

In the early 2000’s, a collection of specimens made by the late Harry Conley from the reefs and rubble beds around the island of Guam, in the western Pacific, were presented by Gustav Paulay (then of the University of Guam) to the second author for study. This resulted in some important publications contributing to the knowledge on the brachyuran fauna of Guam and the Mariana Islands (Ng 2002a, b, c; Tan and Ng 2003; Ng and Takeda 2003; Ng and Ng 2003; Paulay et al. 2003; Lasley et al. 2010). However, some of this material has remained unstudied. Among them were two small and unusual specimens which are here described as a new genus and a new species assigned to the Christmaplacidae.

## Material and methods

The present material was collected by excavating the rubble at low tide, to a depth of approximately 1–2 m. As noted in Ng (2002b) and Ng and Ng (2003), the fauna in deep rubble (described as chalicophilous by Ng and Takeda 2003) is poorly known, and the collector, the late Harry Conley, had the habit of digging deep into the shallow water rubble fields in Guam, sometimes to depths of 30 m, in his search for shells. In the process, he uncovered many rare and novel species of brachyurans (see also McLay 2001).

Measurements are written as carapace maximum width × carapace median length, in millimeters. Material examined are deposited in the Queensland Museum, Brisbane, Australia (QM) and Zoological Reference Collection of the Lee Kong Chian Natural History Museum, National University of Singapore
(ZRC). The following abbreviations are used: **coll.** = collected by; **G1**, **G2** = male first and second gonopods, respectively; **P1-P5** = first to fifth pereopods, respectively (P1 being the chelipeds and P2-P5 are the first to fourth ambulatory legs); **stn.** = station. The sutures on the thoracic sternum are referred to by the numbers of the adjacent thoracic sternites separated by a slash (e.g. suture 5/6 is the suture between sternites 5 and 6). Terminology essentially follows that of Mendoza and Manuel-Santos (2012), Mendoza et al. (2012), and Naruse and Ng (2014).

## Taxonomy

### Superfamily Pseudozioidea Alcock, 1898

#### Family Christmaplacidae

Taxon classificationAnimaliaDecapodaChristmaplacidae

Naruse & Ng, 2014

##### Diagnosis.

Carapace subovate; anterior half of anterolateral margin arcuate, cristate, granulate, posterior half armed with two widely spaced sharp teeth. Eyes reduced, immobile; orbits with sunken exorbital angle, with strong, ridged, anteriorly projecting infraorbital tooth (inner orbital angle), the mesial surface of which receives the antennule. Antennules elongated, cannot be fully retracted into their fossae. Cheliped with large, sharp, triangular, lobiform inner carpal spine; merus with highly convex flexor margin lined with conical spines; major chela with modified opposing molariform teeth on proximal cutting margins of fingers. Ambulatory legs elongate, very slender, meri at least five times as long as wide. G1 sinuous, without spiniform granules; G2 short, about one-third length of G1, distal segment short, about one-fifth total length of G2, petaloid in shape (emended from Naruse and Ng 2014).

#### Harryplax gen. n.

Taxon classificationAnimaliaDecapodaChristmaplacidae

http://zoobank.org/C1615819-9D6D-402A-B6AC-4084BFC28EC0

##### Type species.

*Harryplax severus* sp. n., by present designation.

##### Diagnosis.

Carapace transversely subovate; dorsal surface granular, regions poorly defined; front bilobed, produced beyond orbits; anterolateral margin arcuate, cristate, lined with granules, with two teeth after exorbital angle; posterolateral margins straight, converging posteriorly; endostomial ridge strongly developed. Antennules well developed; second and third articles relatively long, stout, partially retractable into antennular fossa, distal tip of second article reaching infraorbital tooth; basal article of antenna rectangular, slender (much longer than wide), subsequent two articles elongate, flagellum long. Thoracic sternum narrow, granulate; thoracic sternites 1 and 2 fused, triangular; thoracic sternite 3 demarcated from sternite 2 by distinct transverse suture; thoracic sternites 3 and 4 nearly fused except for notches restricted laterally, which continue medially as oblique grooves, forming wide V, and forming the boundary between the two sternites; sternite 4 long, tip of male telson not reaching level of P1 condyles when pleon folded against thoracic sternum; male press-button present as rounded tubercle on sternite 5, midway between sutures 4/5 and 5/6. Median line present on exposed portion of sternite 4, absent in sterno-pleonal cavity except at level of sternites 7 and 8. Penis protruding from gonopore anterior to coxo-sternal condyle of P5. Vulva large, on sternite 6 abutting against posterior border of sternite 5, sub-circular operculum present. Chelipeds robust, distinctly asymmetric, not exhibiting any sexual dimorphism; major chela with eroded molariform tooth on proximal cutting margin of dactylus, large molariform tooth on proximal cutting margin of fixed finger; carpus with broadly triangular, sharp tooth on inner margin; merus anterior margin lined with conical spines. Ambulatory legs long, slender; anterior margins lined with small spines. Male pleon relatively broad; all somites and telson freely articulated. G1 slender, slightly sigmoid, surfaces without spines or sharp granules, distal half lined with stiff, short simple setae; G2 stout, about one-third of G1 length, distal segment short, petaloid.

##### Etymology.

The new genus is named primarily in honor of the intrepid field collector, the late Harry T. Conley, who collected many interesting crustaceans in the rubble beds of Guam, including the species presently being described. The name is also an allusion to a famous namesake, Harry Potter, the magical hero of the popular book series by J.K. Rowling, and Mr. Conley’s uncanny ability to collect rare and interesting creatures as if by magic. The name is an arbitrary combination of “Harry” and the suffix “-plax”. Gender feminine.

##### Remarks.

*Harryplax*, new genus, is classified in Pseudozioidea following Naruse and Ng’s (2014) definition based on the following morphological features: 1) having all the somites of the male pleon and the telson freely articulated; 2) the G2 being about a third the length of the G1; 3) the penis emerging from a coxal (P5) gonopore that is anterior to the coxo-sternal condyle; and 4) the large vulvae which are positioned close to each other in the thoracic sternum. Naruse and Ng (2014) exhaustively discussed the comparative morphology of the pseudozioid families, viz. Pseudoziidae, Planopilumnidae, Pilumnoididae, and Christmaplacidae (see also Guinot and Macpherson 1987; Davie 1989; Ng and Wang 1994; Ng and Liao 2002; Ng 2010; Ng and Ahyong 2013). They highlighted the diagnostic characters of Christmaplacidae which effectively distinguish it from the rest of Pseudozioidea as follows: “The immobile eyes without pigmentation, elongated antennules that cannot retract into their fossae, large lobiform inner carpal spine of the cheliped, large lobiform and spiniform flexor margin of the merus of the cheliped, and the elongated ambulatory legs are unique characters in the Pseudozioidea.” (Naruse and Ng 2015: 270, Table 1).

*Harryplax* is assigned to Christmaplacidae as it shares the following features with the type genus, *Christmaplax*: 1) a sub-ovate carapace with a strongly arcuate anterolateral margin armed with two well-spaced teeth after the effaced exorbital angle; 2) reduced and immobile eyes; 3) robust and similarly proportioned chelipeds, where the major chela has the two opposing modified teeth on the proximal cutting margins of the fingers; 4) long, slender ambulatory legs (unique in Christmaplacidae); 5) a sternal press-button equidistantly positioned on sternite 5 between suture 4/6 and 5/6; 6) a penis emerging from a gonopore on the P5 coxa, anterior to its coxo-sternal condyle; and 7) a simple unarmed G1 with a relatively short and stout G2, the terminal segment of which is short and petaloid in shape.

**Figure 1.**
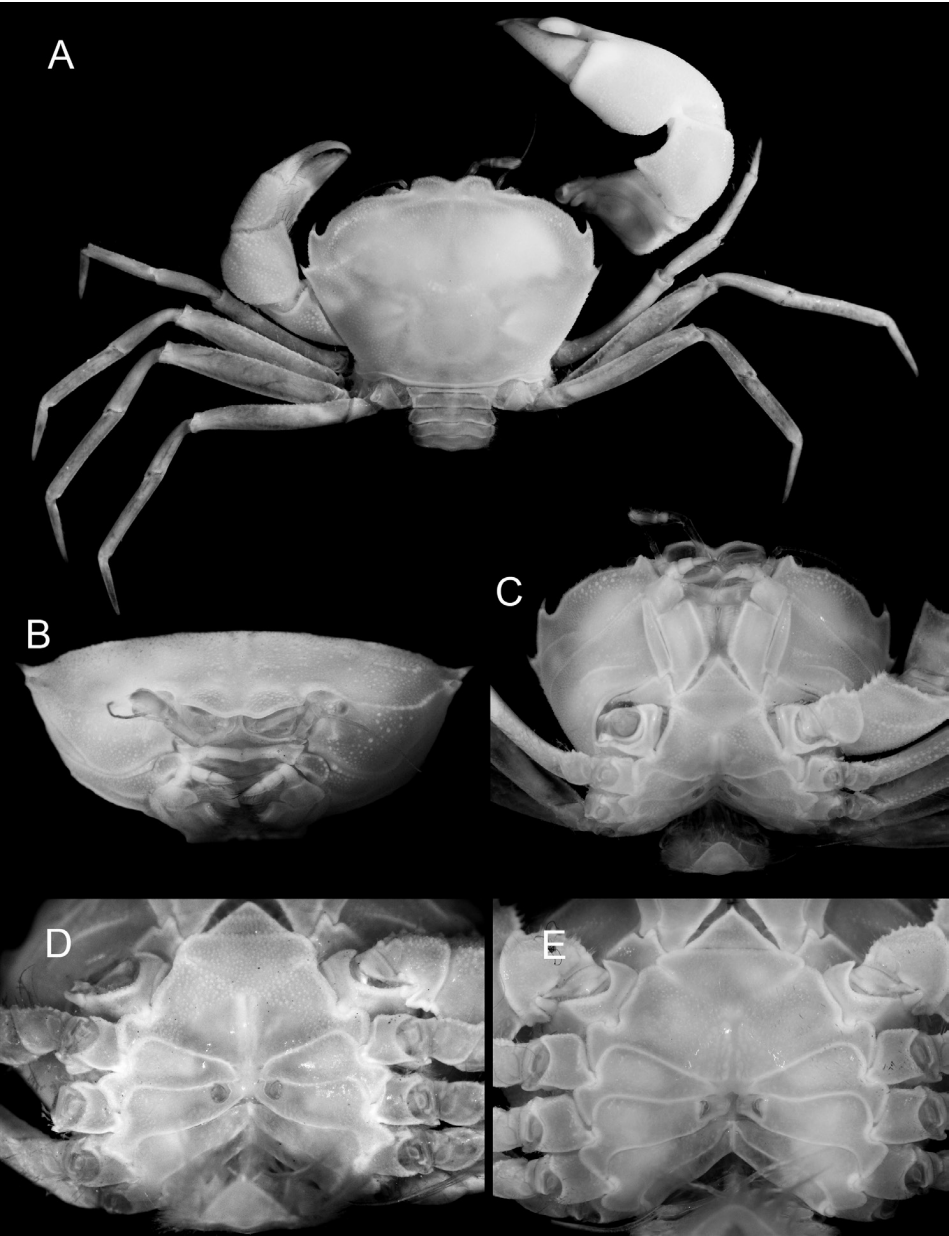
**A–D** *Harryplax severus* gen. & sp. n., holotype, female (ZRC 2016.0253) **E** *Christmaplax mirabilis* Naruse & Ng, 2014, paratype, female (ZRC 2014.0814) **A** habitus, dorsal view **B** cephalothorax, frontal view **C** cephalothorax, ventral view **D, E** thoracic sternum and vulvae, ventral view.

**Figure 2.**
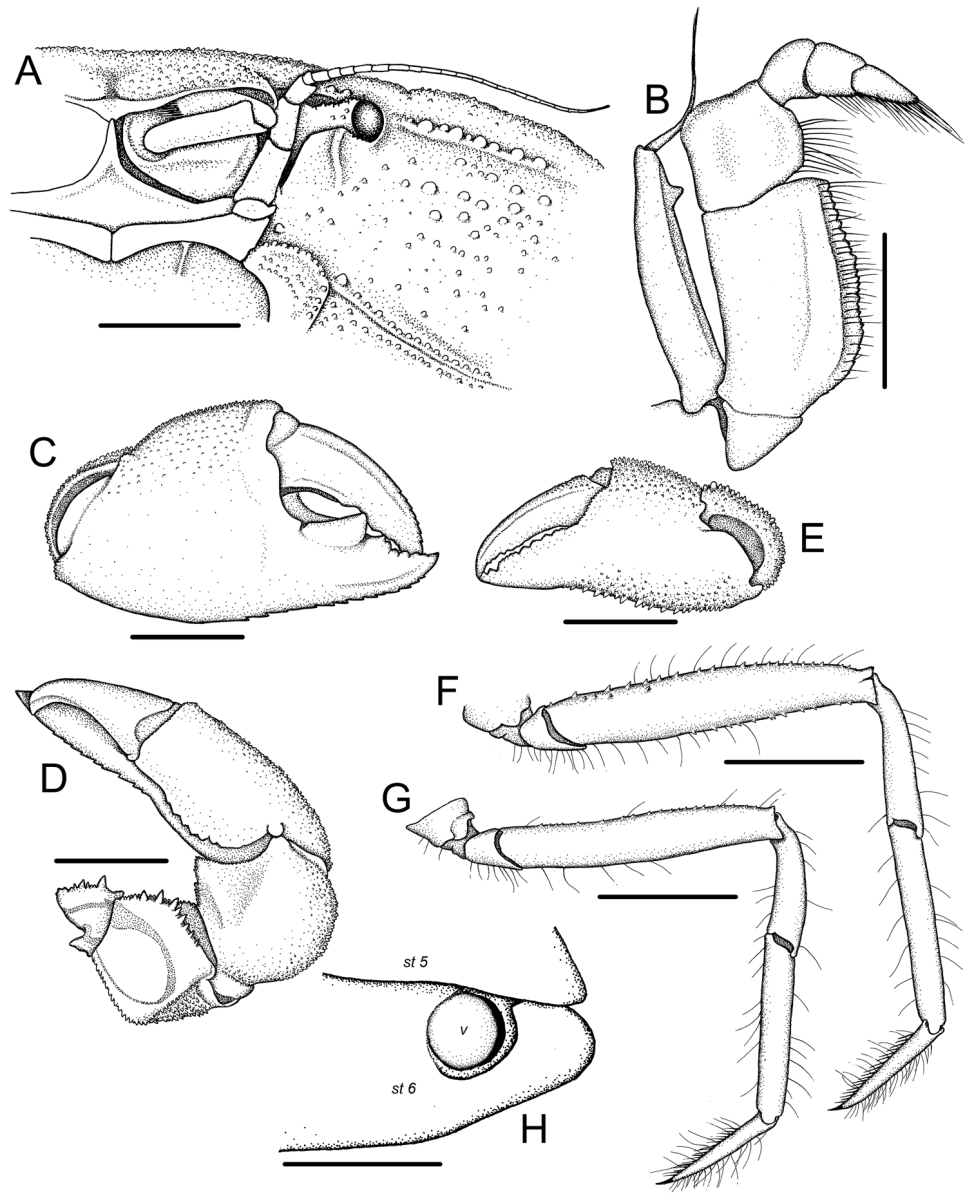
*Harryplax severus* gen. & sp. n., holotype, female (ZRC 2016.0253). **A** cephalothorax, left side, antero-ventral view **B** right third maxilliped, ventral view **C** right (major) chela, external view **D** right (major) cheliped, dorsal view **E** left (minor) chela, external view **F** right P4, dorsal view **G** right P5, dorsal view **H** right vulva, ventral view. Legend: st 5, 6 – sternites 5 and 6, respectively; v – vulva. Scale bars: 1.0 mm (**A, B**), 2.0 mm (**C–G**), 0.5 mm (**H**).

**Figure 3.**
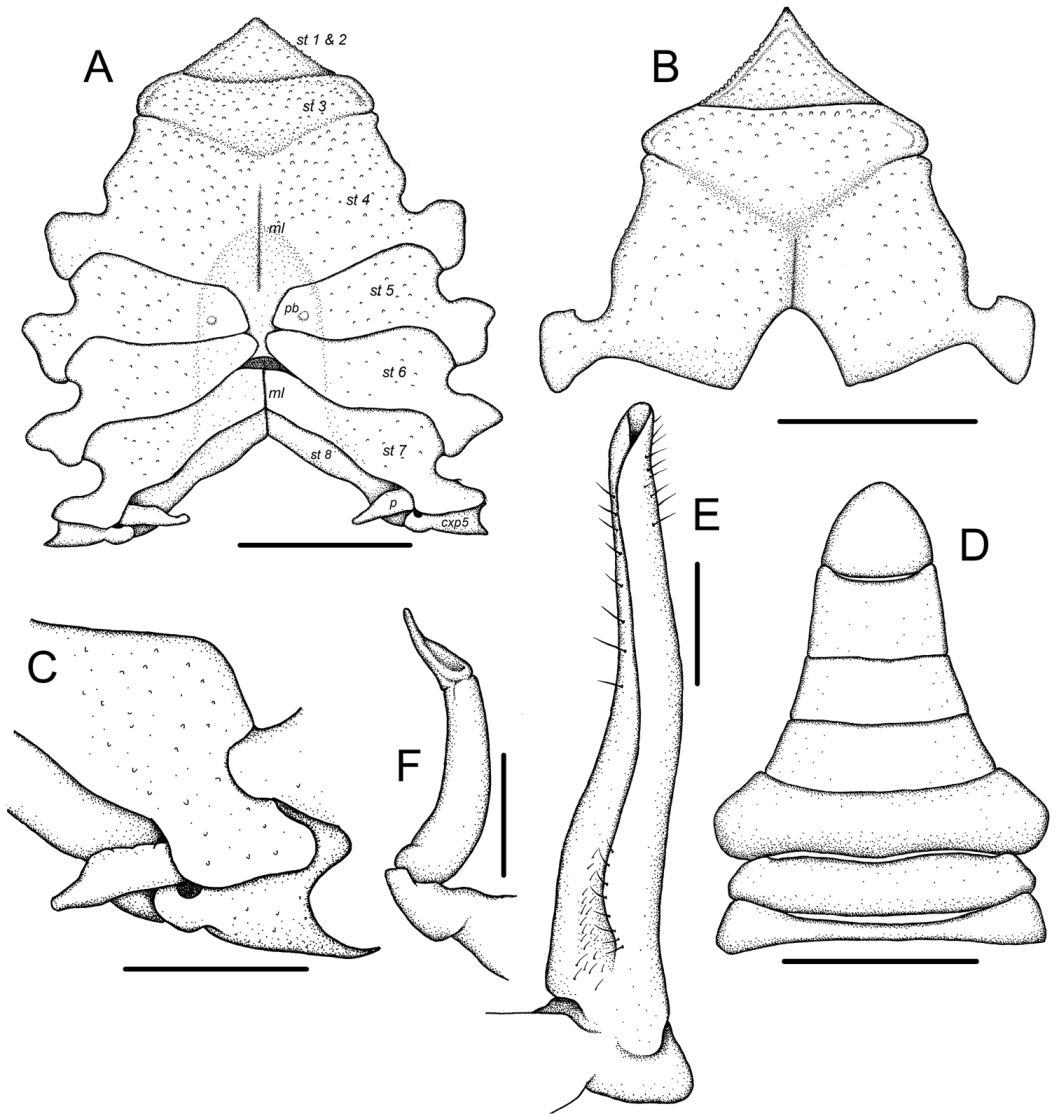
*Harryplax severus* gen. & sp. n., paratype, male (ZRC 2016.0154). **A** thoracic sternum (sternites 1–8), ventral view **B** anterior thoracic sternum (sternites 1–4), ventral view **C** posterior sternites & penis, posteroventral view **D** pleon, ventral view **E** left G1, external view **F** left G2, external view. Legend: cxp5 – coxa of P5; ml – median line; p – penis; pb – tubercle of the sterno-pleonal press-button; st 1–8 – sternite 1–8, respectively. Scale bars: 1.0 mm (**A, B, D**), 0.5 mm (**C**), 0.25 mm (**E, F**).

There are several morphological features, however, that distinguish *Harryplax* from *Christmaplax*:

The carapace has the front more distinctly projecting beyond the supraorbital margin in dorsal view (Fig. 1A) (front only slightly projecting; cf. Naruse and Ng 2014: figs 1a, 2b, 3a);

There are no distinct notches separating the supraorbital margin from the front nor from the anterolateral margin (Fig. 1A) (slight notch between front and supraorbital margin, and deep, pronounced notch marking boundary with anterolateral margin; cf. Naruse and Ng 2014: figs 1a, 2b, 3a);

The anterior portion of the anterolateral margin is more gently arcuate (less convex) but is more strongly cristate (Figs 1A, B, 2A) (more strongly convex and with the crest less pronounced; cf Naruse and Ng 2014: figs 1a-c, 2a, 3a);

The teeth on the carapace anterolateral margin, especially the first, are more prominent, with the tips distinctly curved (Fig. 1A) (teeth relatively smaller with weakly curved tips; cf. Ng and Naruse 2014: figs 1a, 3a);

The eyes, while immobile and reduced, are relatively better developed with longer peduncles and bigger corneas, and are visible even from dorsal view (Fig. 1A, B, 2A) (hidden from dorsal view, sunken into orbit, with shorter peduncles and much reduced corneas; cf. Naruse and Ng 2014: figs 1a, c, 2a, b);

The basal article of the antenna is much longer and narrower (Fig. 2A) (shorter and wider; cf. Naruse and Ng 2014: figs 1c, 6a);

The second and third antennular segments are stout (especially the third), and, although the joint between two articles also reaches the mesial surface of infraorbital tooth when folded, the third article can be partially folded into antennular fossa (Figs 1B, 2A) (second and third antennular articles slender, too long to fold into antennular fossa; cf. Naruse and Ng 2014: fig. 2a, b);

The thoracic sternum is relatively narrower and the posterior end of sternite 3 is slightly wider than the anterior end of sternite 4 (Figs 1C, D, 3A, B) (thoracic sternum distinctly wider, posterior end of sternite 3 distinctly narrower than anterior end of sternite 4; cf. Fig. 1E; Naruse and Ng 2014: figs 1b, 3b, 6c);

Thoracic sternite 4 has a distinct median line in both the male and the female (Figs 1C, D, 3A, B) (median line absent on sternite 4; cf. Fig. 1E; Naruse and Ng 2014: figs 1b, 3b, 6c);

The vulvae are round, and their anterior edge is in contact with thoracic suture 5/6 (Figs 1D, 2H) (vulvae lunate, 'half-moon', in shape, anterior edge not touching suture 5/6; cf. Fig. 1E; Naruse and Ng 2014: fig. 5d);

The endopod of the third maxilliped is relatively narrower (esp. merus) and the lateral margin of the exopod is convex (Fig. 2B) (endopod broader, lateral margin of exopod straight; cf. Naruse and Ng 2014: fig. 6b).

The ambulatory legs are relatively shorter and stouter, and the dactyli are distinctly shorter than the propodi (Figs 1A, 2F, G) (ambulatory legs longer and more slender, dactyli subequal in length to propodi; cf. Naruse and Ng 2014: figs 1a, 3a);

The G1 is only slightly sinuous throughout its length, and the G2 is relatively stouter (Fig. 3E, F) (G1 distinctly sinuous, more pronouncedly curved, G2 more slender; cf. Naruse and Ng 2014: fig. 5b, c).

Naruse and Ng (2014: 265, 270) observed that there was a transverse ridge separating thoracic sternites 2 and 3 in *Christmaplax mirabilis*, but this is not an accurate description of the structures. What separates sternites 2 and 3 is a prominent transverse suture (as in *Harryplax severus*) but the margin of sternite 2 adjacent to the suture is just slightly raised. The different morphological features of *Harryplax* require the emendation of the current diagnosis of the family Christmaplacidae (see above).

#### Harryplax severus sp. n.

Taxon classificationAnimaliaDecapodaChristmaplacidae

http://zoobank.org/9A33FCD1-E030-4FCD-AA35-F648D1FFE3C4

Figs 1A–D , 2 , 3

##### Type material.

Holotype ♀ (7.9 × 5.6 mm) (ZRC 2016.0253), Guam, Piti Reef margin, 1–1.5 m depth, in rubble; coll. H.T. Conley, September 1998; paratype ♂ (5.4 × 3.9 mm) (ZRC 2016.0254), Guam, Glass Breakwater near mouth of Apra Harbour, 4.6–7.6 m depth, among rocks; coll. H.T. Conley, 18 July 2001.

##### Comparative material.

*Christmaplax mirabilis* Naruse & Ng, 2014, holotype ♂ (11.0 × 7.9 mm) (QM W29223), stn. CI-D04, Christmas Island, Thundercliff Cave, coll. T. Naruse & Y. Fujita, 15 February 2012; paratype ♀ (11.3 × 8.3 mm) (ZRC 2014.0814), stn. CI-D07, same locality as holotype, coll. Y. Fujita & T. Naruse, 16 February 2012.

##### Description.

Carapace (Figs 1A, B, 2A) transversely subovate, 1.38–1.41 times as wide as long, dorsal surface slightly convex, mostly smooth but becoming more granulate at periphery, regions poorly defined; H-shaped gastric grooves barely discernible. Front well produced anteriorly, ventrally deflexed; frontal margin bilobed, lobes separated by wide V-shaped concavity, anterior margins slightly concave, mesial angles more produced than lateral. Supraorbital margin granulate, forming slightly obtuse angle with base of front, continuing uninterrupted into anterolateral margin (without notches); infraorbital margin much shorter, junction with supraorbital margin sunken; orbit small, laterally unarmed, infraorbital angle produced as large ridge-like tooth, mesial surface slightly concave for accommodating distal end of second antennular article when folded, tooth produced anteriorly beyond orbit when viewed dorsally. Exorbital angle not clearly marked in dorsal view. Anterior half of anterolateral margin, arcuate, cristate, lined with round granules; posterior half with two strong teeth with sharp, incurved apices, first tooth distinctly larger, more curved than second tooth which marks junction between antero- and posterolateral margins. Posterolateral margins almost straight, convergent posteriorly; surfaces covered with tiny granules. Suborbital, subhepatic, and pterygostomial regions covered with many fine and some larger granules; pterygostomial region with a granulate ridge anterior to Milne Edwards’ aperture. Epistome short, with medial transverse depression, posterior margin with a small median projection. Endostomial ridge strongly developed. Lateral margins of buccal cavern subparallel, slightly convergent anteriorly, concave.

Antennules (Figs 1B, 2A) well developed; basal antennular segment large, high, upper half forming cavity for second article; second and third articles long, can be partially retracted into antennular fossae, second article longer than third, which has distal end wider than proximal end; mesial surface of internal orbital tooth accommodating joint of second and third antennular articles when folded. Antennal basal article subrectangular, longer than wide, not completely filling orbital hiatus, barely mobile, subsequent two articles elongate, cylindrical; flagellum long, reaching first anterolateral tooth when folded laterally along anterolateral margin.

Eye (Figs 1A, B, 2A) reduced, immovable; visible from dorsal view of carapace, with small granules on dorsal surface; cornea discernible on rounded, bulbous distal end of eyestalk, visible from dorsal and anterior views.

Third maxillipeds (Figs 1B, C, 2B) forming narrow triangular median hiatus when closed; ischium long, median length about twice of that of merus, with shallow longitudinal sulcus; merus quadrate, anterior margin slightly concave, distolateral angle rounded, distal and mesial margins granulate; exopod slender, lateral margin convex, mesial margin with subdistal triangular tooth, flagellum long.

Male thoracic sternum (Fig. 3A, B) transversely narrow. Sternites 1 and 2 fused, triangular in outline, apex acute, lateral margins straight; sternite 2 separated from sternite 3 by distinct transverse suture; sternites 3 and 4 almost completely fused except for incomplete sutures laterally which continue mesially as shallow but distinct V-shaped groove; sternite 4 long, narrow, ratio of width (measured at lateral extremes of episternites) over length (measured as distance between tip of closed telson to center of suture 3/4) = 3.5; male pleonal locking mechanism (press-button) present as round tubercle on sternite 5 midway between sutures 4/5 and 5/6; sutures 4/5 and 5/6 interrupted medially; suture 6/7 fused medially, median marked by trapezoidal area that is less calcified than surrounding area of sterno-pleonal cavity, appearing like a ‘hole’; suture 7/8 fused medially and connecting with median line; sternite 7 widely exposed when pleon closed, wider than long; small portion of sternite 8 exposed between lateral edges of pleomeres 2 and 3 when pleon is pressed closed against thoracic sternum. Median line present on exposed region of sternite 4, absent within sterno-pleonal cavity except at level of sternites 7 and 8. Penis (Fig. 3C) protruding from gonopore on P5 coxa, anterior to coxo-sternal condyle. Female thoracic structure (Figs 1C, D) similar to that of male; vulva (Fig. 3H) on sternite 6, round, relatively large, anterior border contacting sture 5/6, operculum similarly round, no sternal projections.

Chelipeds (Figs 1A, 2C–E) distinctly asymmetrical, right chelipeds more robust and specialized in examined material; no obvious sexual dimorphisms. Anterior margins of basis-ischium and merus of both chelipeds lined with sharp conical spines of varying length, upper margin of merus distinctly convex, cristate, lined with pointed granules, lower outer margin gently convex, cristate, granulate, terminating distally with granulate tuberosity. Carpus finely granulate, with strong, sharp, broadly triangular, lamellar tooth on inner margin (larger in major cheliped). Major chela wide, inflated; external and internal surfaces mostly smooth; upper surface weakly granulate, inner margin of upper surface carinate, with transverse concavity immediately beneath it on inner surface; lower margin lined with sharp granules. Fingers stout, fixed finger shorter than movable finger, almost straight except for upcurved tip, cutting margin with large, proximal molariform tooth with flattened occlusal surface, followed distally by 2 smaller cutting teeth, lower margin armed with large conical granules; movable finger gently curved downwards, with large, but eroded, flat tooth proximally, followed distally by 1 cutting tooth. Minor chela relatively narrower, more granulate, fingers less robust, without molariform teeth, lower margin with conical spines.

Ambulatory legs (Figs 1A, 2F, G) long, slender, sparsely setose; P4 longest, combined merus-to-dactylus length 1.57–1.60 times carapace width, merus approximately five times as long as wide; P2 and P5 shortest. Meri flattened, margins lined with sharp granules, which are much smaller on P5; other articles unarmed, except for propodus of P2 which is lined with small sharp granules; carpus subcylindrical, curved proximally; propodus straight, flattened; dactylus subcylindrical, tapering distally, terminating in sharp curved claw, flexor margins with row of closely packed short stiff setae.

Male pleon (Fig. 3D) with all somites and telson free; somites 1 and 2 short; somite 1 partially concealed under posterior margin of carapace, with transverse ridge; somite 3 widest, subsequent somites trapezoidal, progressively narrowing, producing a combined lateral margin that is concave; telson subtriangular, lateral margins convex, apex rounded. Female pleon longitudinally oval with all somites and telson free; relatively narrow, somite 3 widest, telson wider than long; in female holotype, pleopods developed, setose throughout entire length.

G1 (Fig. 3E) slender, slightly sinuous, unarmed, mesial and lateral margins each with row of stiff simple setae towards distal end; aperture terminally placed. G2 (Fig. 3F) about one-third length of G1, distal segment petaloid in shape.

##### Etymology.

The specific epithet, *severus* (L., harsh, rough, rigorous), alludes to the rigorous and laborious process by which this crab was collected. It is also an allusion to a notorious and misunderstood character in the Harry Potter novels, Professor Severus Snape, for his ability to keep one of the most important secrets in the story, just like the present new species which has eluded discovery until now, nearly 20 years after it was first collected. The name is used here as a noun in apposition.

##### Remarks.

*Harryplax severus* sp. n., shares some key morphological features with *Christmaplax mirabilis* which are presumably adaptations to a stygobitic lifestyle (i.e. reduced eyes, well-developed antennules and antennae, and long, slender ambulatory legs), which in turn suggests that the environmental conditions under which the former thrives are probably similar to the conditions in underwater caves. *Harryplax severus* is clearly a chalicophilous species, as it was collected deep in coral rubble or under subtidal rocks. It is also possible that it is a cavity dweller, or coelobite (viz. Choi and Ginsburg 1983; Choi 1984; Takada et al. 2007, 2008), living within the interstices of coral rubble and rocks. It is clearly a part of the poorly known coral reef cryptofauna and its reclusive nature accounts for its rarity and absence in conventional reef surveys. The new species is only known from the type locality, Guam, thus far.

## Supplementary Material

XML Treatment for Christmaplacidae

XML Treatment for Harryplax

XML Treatment for Harryplax severus
